# The Creation and Statistical Evaluation of a Deterministic Model of the Human Bronchial Tree from HRCT Images

**DOI:** 10.1371/journal.pone.0168026

**Published:** 2016-12-15

**Authors:** Spyridon Montesantos, Ira Katz, Marine Pichelin, Georges Caillibotte

**Affiliations:** 1 Medical R&D, Air Liquide Santé International, Paris Saclay, France; 2 Department of Mechanical Engineering, Lafayette College, Easton, PA, United States of America; Technion Israel Institute of Technology, ISRAEL

## Abstract

A quantitative description of the morphology of lung structure is essential prior to any form of predictive modeling of ventilation or aerosol deposition implemented within the lung. The human lung is a very complex organ, with airway structures that span two orders of magnitude and having a multitude of interfaces between air, tissue and blood. As such, current medical imaging protocols cannot provide medical practitioners and researchers with in-vivo knowledge of deeper lung structures. In this work a detailed algorithm for the generation of an individualized 3D deterministic model of the conducting part of the human tracheo-bronchial tree is described. Distinct initial conditions were obtained from the high-resolution computed tomography (HRCT) images of seven healthy volunteers. The algorithm developed is fractal in nature and is implemented as a self-similar space sub-division procedure. The expansion process utilizes physiologically realistic relationships and thresholds to produce an anatomically consistent human airway tree. The model was validated through extensive statistical analysis of the results and comparison of the most common morphological features with previously published morphometric studies and other equivalent models. The resulting trees were shown to be in good agreement with published human lung geometric characteristics and can be used to study, among other things, structure-function relationships in simulation studies.

## Introduction

Modeling lung function is a field of increasing research intensity. Such models are currently used for, among other things, the study of aerosol deposition [1–9], gas and vapor flow mechanics [8,10–13], the distribution of ventilation in various conditions of health and disease [14–17] and the analysis of medical images for either diagnostic or patient-treatment purposes [4,6,18–21]. However, the meaningful interpretation of all the above presumes knowledge of the physiological and geometric characteristics of the bounding structure within which the gas flows, i.e. the tracheo-bronchial tree. Therefore, a quantitative description of the morphology of this structure becomes essential prior to any other form of predictive modeling implemented in the lung.

The human lung contains a complex multi-scale range of interfaces between air, tissues and blood such that it is only possible to obtain the morphology of the central airways through the use of medical images. For that reason and depending on the application, a series of models based on experimental measurements on excised lungs have been proposed [1,3,15,22–25]. Furthermore, with the help of improved computational techniques, 3D models simulating the bifurcating structure of the bronchial tree down to various depths were created. Martonen et al. [26] were the first to generate such a model using a symmetric expansion of the branching structure using diameter, length and branching angle data from [22] and 90° rotations. Kitaoka et al. [27] took advantage of a relationship between diameter and flow to generate a more realistically asymmetric tree-like structure. Their model also takes into account the general shape of the confining space, i.e. the lung. However, this model is considered to be more asymmetric than real lungs while not enough data are available concerning the basic morphometric features of the generated tree.

To our knowledge, the most effective model of the human airway tree was presented by Tawhai et al. [28]. The process was, in essence, a volume-dividing algorithm. The goal was to sub-divide the lung space in a computationally efficient way while simultaneously generating the tree branches that supplied each sub-space in a self-similar manner. This principle as a solution for the description of biological bifurcating systems was first described by Mandelbrot [29], who proposed an algorithm for an area-filling fractal branching system to describe 2D river drainage basins. A simplified technique using a Monte Carlo algorithm was later suggested by Wang et al. [30] and was revised into 3D by [28] for the purpose of creating models for mammalian tracheo-bronchial trees. Their original model was developed into a confining space formed from the segmented lung volumes from Magnetic Resonance Images (MRI). A grid of uniformly spaced seed points was calculated to lie within each lobe, confined by the relevant surfaces. The central airways down to the lobar bronchi were taken from the literature [23] and adapted to fit the MRI data, with the lobar bronchi forming the starting points for the development of the algorithm. In a later validation study [31], the confining surfaces and detailed airway tree were obtained through segmentation of Multi-Detector Computed Tomography (MDCT) of the human and ovine lungs. A similar technique was used in our study, utilizing the voxels of the segmented HRCT images to generate a much denser seed dataset. In a later validation paper, medical imaging data from a single human volunteer were used instead of the generalized lung atlas information employed in the first paper [31]. Recently, Bordas et al. [32] proposed a work pipeline for the generation of such models and validated them using measured morphometric data and calculated airway resistances in healthy and asthmatic patients.

The current study proposes an alternative work-flow pipeline to that of [32]. The algorithm created by Tawhai et al. [28] was implemented with certain modifications. Algorithm expansion parameters were formulated and described using morphological information from the literature and implemented with medical image data from human volunteers to provide a set of airway tree models for healthy humans. The ensuing models were validated based on key lung morphology parameters found in the literature.

## Methods

### Medical Image Acquisition

The HRCT images were acquired as part of a study at the University Hospital Southampton NHS Foundation Trust (clinical trail EudraCT #2007-003563-43). The study is described in detail elsewhere [19,33]. Approval was obtained from the local research ethics committee and the UK Administration of Radioactive Substances Advisory Committee. In brief, the study included seven healthy subjects and six patients with moderate persistent asthma (GINA guidelines 2005) free from exacerbation for at least four weeks. All subjects were male, non-smokers between the ages of 20 and 58. The healthy subject demographic information can be observed in Table 1.

**Table 1:** Demographic information.

	Gender	Age (years)	Height (cm)	Weight (kg)	FEV1%	FEV1/FVC %	FRC (ml)	CT Air vol. (ml)
H01	M	24	174	82	107	98	2360	1905
H02	M	20	187	88	115	103	4800	5829
H03	M	26	177	78	102	99	3170	3857
H04	M	20	186	87	114	99	4170	3533
H05	M	27	173	80	91	92	3480	4220
H06	M	31	179	82	96	95	2910	3867
P04	M	45.5	173	80	107	106	2800	3595

The FRC was calculated via spirometry in the erect position while the lung air volume was calculated from the HRCT in the supine position.

A Sensation 64 slice HRCT scanner (Siemens, Erlangen, Germany) was utilized to acquire 3D images from the mouth to the base of the lungs. The acquisition was performed with the subject in the supine position during slow exhalation against a resistance after a tidal breath. This maneuver was intended to image the glottal aperture in the open position. The average scan time was 12.3 s. The imaging parameters were 120 kVp, 120 mAs and a pitch of 1.0. The average effective dose of radiation for each subject was estimated to be 4.5 mSv. The captured 3D images have slice thickness of 0.75mm, with pixel spacing varying between 0.55 and 0.65mm and slice separation of 0.5mm. The data are stored in DICOM format, with image matrices of typical size 512x512x650.

### Software for Bronchial and Lung Geometry Acquisition

The initial information used for the development of the airway tree model were the geometric characteristics of the “real” bronchial tree as determined from the medical images obtained in the clinical study. The Pulmonary Workstation 2 (PW2) software package (VIDA Diagnostics, Coralville, IA) was used for the segmentation and measurement of the airway tree bronchi down to the most distal distinguishable generation. This was typically generation 3–4 in the upper lobes and generation 6–7 in the lower lobes. The measurements include all the morphometric attributes of the airways. These are essential for the reconstruction of the bronchial tree as a network of interconnected straight tubes and include average lumen diameters, airway lengths, branching and rotation angles, branch start and end coordinates, etc. The lung and lobar volumes were also segmented to provide our algorithm with the necessary confining spaces. The manual and semi-automatic use of the PW2 software package as a research tool is explained in [34]. The airway tree model itself was developed using MATLAB v8.4 (R2014b) (The Mathworks Inc, Natick, MA).

### Pre-processing and Initial Information

As mentioned earlier, the initial conditions for the model can be obtained through anatomical atlases or through analysis of individual medical images. The availability of HRCT images in this study allowed us to define individualized and anatomically consistent initial conditions. The pulmonary lobes were selected as confining spaces. Due to the nature of the data, the voxels of the lobar volumes can be used to generate the grid of seed points. The created set of seeds was unnecessarily dense and does not represent the number of acini in the lung; it was therefore sub-sampled to form a more manageable grid, maintaining however multiple seeds per acinus. The number of seeds per acinus is calculated with the help of the voxel linear dimensions, with a minimum value of 1. If computer memory and power considerations are taken into account, a typical sub-sampling down to 32–64 voxels per seed is sufficient for the creation of a robust tree.

The basis for the tree model is also formed with the help of the segmented bronchi from the medical images. Since the segmentation is performed to the most distal airway possible, it is necessary to implement an initial division into sub-volumes; these are assigned to the ‘real’ branches penetrating the confining space. This was done iteratively; starting from the first generation distal to the lobar bronchi, the entire sub-tree attached to each airway is calculated. Every sub-tree is then assigned to a unique sub-space with reference to their distance from the seeds. The process continues along every successive sub-tree, each time dividing the grid to smaller groups until the ‘leafs’ of the segmented structure are reached. These ‘leafs’ and their respective sub-grids form the starting points for the model algorithm. This method ensures the expansion of the model into non-overlapping regions, thus negating the need for additional checks on whether the model branches are developed within their expected spaces.

### Model Expansion Rules and Stopping Conditions

The goal of the model is to recreate the conducting part of the human respiratory tree. As such, the model needs to expand from its starting points down to the terminal bronchioles (TB), where each acinar region begins [28]. A terminal bronchiole is defined by its length and the size of the sub-volume it supplies. Other rules concerning the geometry of the generated tree are also imposed to obtain a more realistic model.

#### Selection of Splitting Plane

The most important decision for the translation of the 2D technique into 3D is the selection of the appropriate volume-dividing plane. The equation giving the coordinates (x, y, z) of a point on the plane is:

$$a(x-x_{CM})+b(y-y_{CM})+c(z-z_{CM})=0,$$ (1)

with the plane normal $\overset{\underset{}{\to }}{norm}=\left[\begin{array}{c} a\\ b\\ c \end{array}\right]$ extending from any known plane point *CM* = (*x*_*CM*_,*y*_*CM*_,*z*_*CM*_).

The following combination of data was selected for the formation of this plane:

1. The normal vector of the plane is defined as the vector perpendicular to the plane of the parent and the parent sibling branches, i.e. $\overset{\underset{}{\to }}{norm}=\vec{P}v\times \vec{P}sv$, where $\vec{P}v$ is the parent vector and $\vec{P}sv$ is the parent sibling vector.
2. The plane point used is the space centre of volume CM.

**Fig 1:**
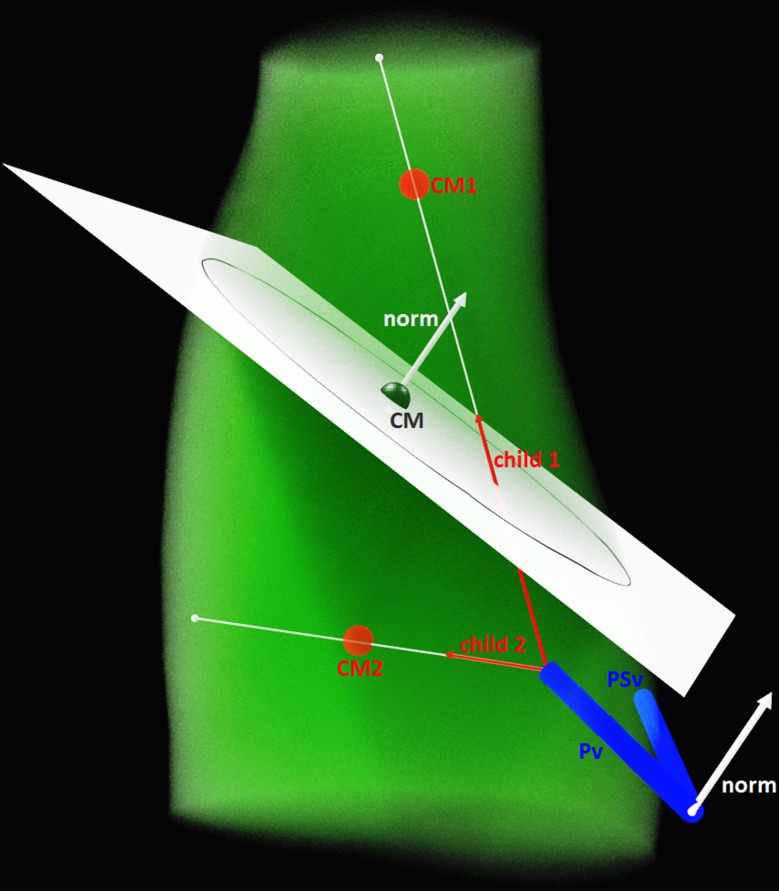
The splitting plane and its elements. CM, CM1 and CM2 are the centres of mass of the volume and its sub-volumes, Pv and PSv the parent and parent sibling vectors, norm is the plane normal vector and child1 and child2 the two daughters extended along the lines passing through the centres of mass toward the edges of the lung.

A single volume sub-division is displayed in Fig 1. The algorithm is iterative and continues until certain conditions are met; these are determined by the general morphometric/geometric realities of the physical system described (e.g. the human lung).

#### Airway Length

In general, branch lengths decrease descending into the lung. However, this physical observation is not directly imposed in determining the length of the model branches. Instead, the length of the created airways is set at 40% of the distance between the parent end-point and the sub-space centre of volume following Tawhai et al. [28].

A lower limit is also imposed on the bronchial length; all generated branches below this are considered to be terminal bronchioles (TB). As the values of reported TB lengths vary considerably in the literature (between 0.65–2 mm) [22,24,35–38], the lower limit was set to 1.4mm.

A final condition is also imposed so that the length of the daughter airways cannot become much larger than the parent length. Thus, the constraint

$$L_{child}\leq 1.5*L_{parent}$$ (2)

was imposed, where L is the length.

#### Airway Diameter

Branch diameters also decrease descending into the lung. The parent-child diameter ratio, known as the Homothety Ratio (HR), was reported in the literature [22,23,31] as

$$D_{child}=2^{-0.33}D_{parent}=0.794D_{parent},$$ (3)

where D is the diameter. Furthermore, a relationship connecting the length and diameter of an airway was investigated in the literature [34,39] and was summarized by Sauret et al. [40] as the rule of thumb,

$$\frac{L}{D}=3\pm 1.$$ (4)

To avoid creating an overly symmetric structure, Eq 4 was used to select tube diameters in lieu of the HR such that D = L/3. A diameter lower limit with respect to TB sizes was also considered; however, since the tree expansion process is not dependent on the diameter, no such limitation was imposed.

Finally, a higher constraint to the child airway diameter with respect to its parent was imposed:

$$D_{child}\leq 0.95*D_{parent}.$$ (5)

#### Branching Angle

The branching angle θ is defined as the angle formed between the parent airway direction and the child airway direction. Because of the irregular shape of the confining space and its subdivisions, the centers of volume can be positioned so that the resulting branch angle is highly variable, with θ ranging between 5°-80° [34]. However, θ is usually below 60° [41] and an optimal value has been calculated at 37.28° based on fundamental physical principles [41–44]. To avoid the possibility of unnaturally large angles, a maximum limit is set at 75°. When the threshold value is exceeded, the model branch is rotated on the plane formed by the branch and the parent so that the angle is reduced to 75°; this is followed by the recalculation of the assigned confining space.

#### Acinar Volume

Not very much direct data concerning the morphology of the human pulmonary acinus exist in the literature. This is mostly due to the difficulty in collecting data in-vivo while the collection of information through lung casts is tedious and prone to errors. Hansen and Ampaya [45] working on the cast of an enlarged reconstructed human acinus measured the volume to be V_ac_ = 182mm^3^, a result confirmed by [35] while working on the silicon rubber casts of the right upper lobe of two human lungs. The latter found V_ac_ to vary between 51 and 459mm^3^, with an average V_ac_ = 187±79mm^3^ distributed in the manner of slightly skewed log-normal distribution. More recently, [37] performed a geometric analysis of the acinus ex-vivo using synchrotron-based micro-CT, finding V_ac_ = 131.3±29.2mm^3^ but also suggesting the possibility of a 20% volume loss due to the formaldehyde preparation of the organ prior to imaging. Finally, [46] calculated V_ac_ = 160±29mm^3^ based on micro CT measurements of alveolar volume and density in excised frozen lungs.

In order to simulate the lung physiology, and considering the fact that the lung volumes of the subjects participating in this study are between functional residual capacity (FRC) and total lung capacity, a threshold was set to the volume of the sub-divided space equal to 25% higher than the value found by [35], i.e. 233.75mm^3^. When a model branch penetrates a generated volume below this threshold, the branch is considered a TB and the expansion of the model along this path is terminated. Even though this threshold does not represent the entire range of values as given by [35,37,38,45], it was assumed that when combined with the other conditions it would provide realistic results.

### Algorithm Description

**Fig 2:**
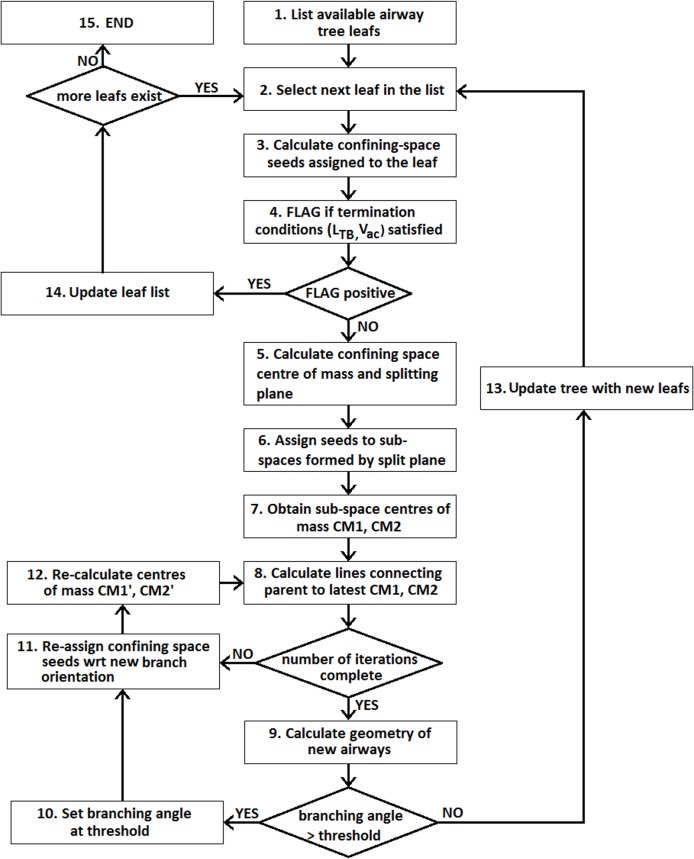
Flow chart of the algorithm.

A flow chart of the algorithm can be seen in Fig 2. The term leaf will be used from this point forward interchangeably with TB. Very briefly, the main steps are the following:

Steps 1–4: For a leaf in the airway tree, a check is performed to see if it fulfills the algorithm termination criteria. A branch is a TB if its length is smaller than 1.4mm and it supplies air to a confining space with volume less than 233.75mm^3^.

Step 5: If the termination FLAG is negative, the centre of volume of the confining space is found and the splitting plane is calculated.

Steps 6–7: The two sub-spaces formed by the splitting plane are assigned their related seeds and their respective centres of volume CM_1_, CM_2_ are calculated.

Step 8: The lines connecting the parent branch to the CM_1_, CM_2_ are calculated and the child branches’ skeleton lines (with length set at 40% the distance between the parent end-point and CM_1_, CM_2_) are formed.

Steps 11–12: If the number of iterations is set i>0, the seeds of the confining space supplied by the parent branch are re-assigned to the skeletons lines of the two children (step 8). Steps 8-11-12 are repeated per number of iterations.

Steps 9 to 12: The diameter (D_j+1_) and branching angle (θ) of the child branches are calculated. If θ>75°, the skeleton of the relevant branch is recalculated with θ = 75°, with its vector remaining on the plane defined by the original child and its sibling. Then steps 11, 12, 8 and 9 are repeated (no iterations).

Step 13: The list of tree airways is updated.

Step 14: If the FLAG is positive proceed to the next tree leaf and repeat steps 2 to 13.

Step 15: The algorithm continues until no un-flagged leafs remain.

**Fig 3:**
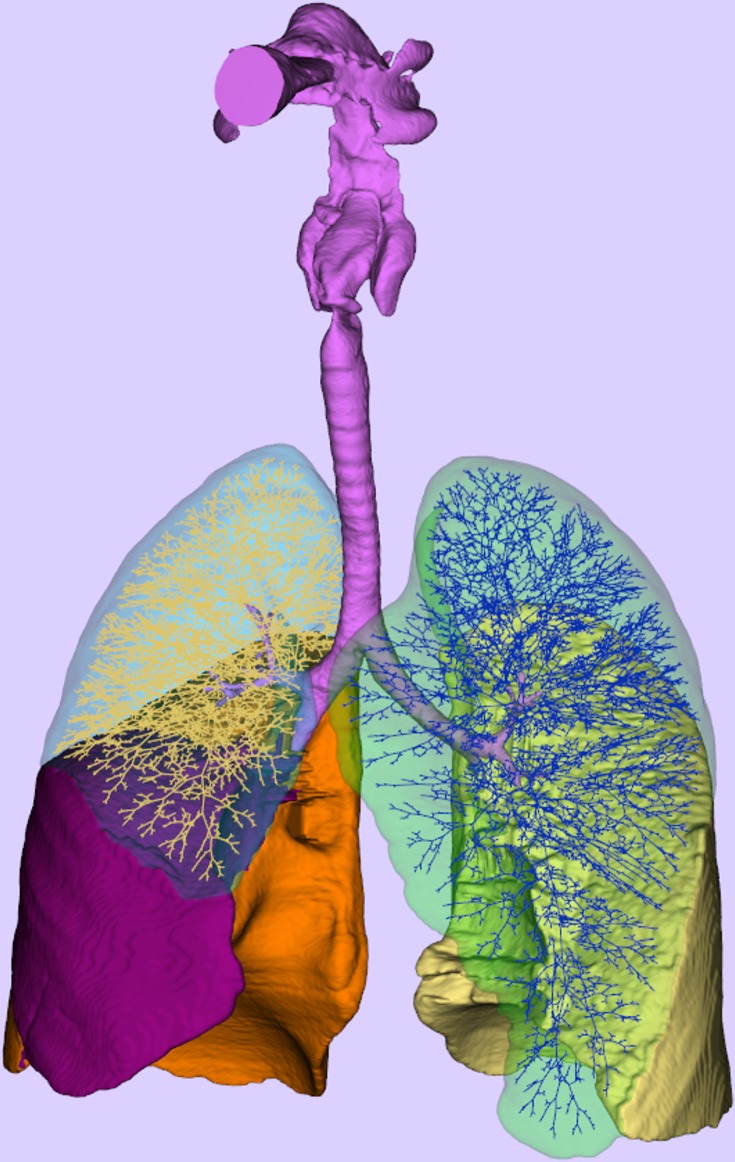
The skeleton representation of the model as it appears when expanded to the edge of the relevant lobar confining spaces.

The results of the above algorithm can be observed in Fig 3.

### Geometric Analysis

Several methods can be used to assemble the airway tree branches with common characteristics under a unique classification system. If the classification of the airways is done with reference to the stem of the tree, i.e. the trachea (generation 0), the branches are grouped by generations, the generation of each branch being one higher than that of its parent [22]. In contrast, if the classification begins at the leafs there are two possibilities. The ordering system proposed by Horsfield et al. [23] assigns order one to the leafs of the system and counts upwards towards the stem by adding one order to the maximum order of each branches’ children. Alternatively, a method first created for the classification of river systems by Strahler [47] and applied by Horsfield et al. [48] to the respiratory system can be used. According to this technique the leafs are again assigned order one and orders are counted upwards toward the stem. However, the order of the parent is one higher than the order of its two children of the same order, or else obtains the order of the highest order child.

Since the model proposed describes the entire conducting part of the airway tree, all three classification systems could be used for airway comparisons. For the determination of generation values, a Boyden-based [49] configuration was utilized for the segmented airways as provided by the segmentation software. A series of measurements was effected concerning all the major geometric characteristics of the generated tree. These included the airway diameters D, lengths L and branching angles θ. Other geometric features measured were the planar rotation angles φ (defined as the angles of rotation between successive bifurcation planes) and relationships such as the Homothety Ratio (HR) and the Length-to-Diameter ratio (L/D). The TB characteristics were treated separately and included the volumes of the calculated acinar regions.

Some structural characteristics of the tree like the average number of generations between the trachea and the TBs were also measured. The most distinctive of those is the branching ratio (RB) and is defined as “the antilog of the slope of the logarithm of the number of branches plotted against the order of branching” [28]. Combined with the Diameter and Length Ratios (RD and RL respectively), these are measures of asymmetry in the branching structure and the rate of decline of diameter and length. For example, a symmetric tree has RB = 2; in a Horsfield ordering scheme RB_H_ decreases as the asymmetry increases while the opposite is true in a Strahler (RB_S_) ordering scheme.

## Results

### Structure and Asymmetry

The general structural information of the model tree is compared with data from the literature in Table 2. The 27763 average number of acini predicted by our model is comparable to those given by [23] and [27], slightly less than [28] and well within the range 26000–32000 as calculated by [35]. However, [45] and [15] indicate a number of acini closer to 23000 while [22] predicted 66000 acini in the human lung.

**Table 2:** Number of acini and generations between trachea and terminal bronchioles.

	No. of Acini	Generation distance of acini from trachea							
		RU	RM	RL	LU	LL	Lungs	min	max
Model	27763.6±7118.5	15.3±1.2	15.2±1.7	17.2±2.2	15.9±1.4	16.3±1.7	16.2±1.7	8	25
Weibel (1963)	66000						16	8	25
Horsfield (1968)	27992						14.6	8	25
Phalen (1978)		15	15	17	15	16	15.6		
Kitaoka (1999)	27706						17.6±3.4	8	23
Tawhai (2000)	29445	16.4	16	17.4	16.3	16.2	16	10	26
Florens (2011)	23000						15–16	9	23

The data are given as mean ± standard deviation (where available) for our model and the following studies, in chronological order of publication: (Anatomical studies) Weibel [22], Horsfield & Cumming [23]. (Model results) Phalen et al. [36]. Kitaoka et al. [27], Tawhai et al. [28] and Florens et al. [15]. Data are provided per lobe where available.

With respect to the number of bifurcations from the trachea that an acinus may appear, termed acinus generation distance (AGR), the model matches well the data of [36] while it agrees with [28] for the RL, LU and LL lobes. For the LU lobe the model prediction is between the other two models. [23] found 1.5 less generations for the termination of the conductive part of the tree while [27] predicts 1.5 generations more. Furthermore, the minimum and maximum AGRs in our model (8 and 25 respectively) compare well with [28] (range 10–26).

The number of branches was plotted against both Horsfield (u_h_) and Strahler (u_s_) orders to calculate RB_H_ and RB_S_ (Table 3). For both quantities, our model seems to be generating slightly more structurally symmetric trees than other models. Especially for RB_S_, our calculated value of 2.49 is marginally lower than the range 2.5–2.8 given by [50,51] and the value of Phalen’s model 2.508 [36], but is much lower than the model of [31] that gives an RB_S_ = 2.8. However, it is higher than the value given by [28] in their original paper describing the model (RB_S_ = 2.358), suggesting that most of the difference in the symmetry of the tree is produced by the utilization of medical imaging data.

**Table 3:** The branching, diameter and length ratios for Horsfield order (RB_H_, RD_H_, RL_H_) and Strahler order (RB_S_, RD_S_, RL_S_) respectively.

	RB_H_	RD_H_	RL_H_	RB_S_	RD_S_	RLs
Model	1.56 (0.98)	1.166 (0.99)	1.13 (0.77)	2.49 (0.99)	1.397 (0.99)	1.392 (0.99)
Phalen (1978)				2.508	1.351	1.333
Horsfield (1981)				2.51–2.81	1.35–1.45	1.33–1.46
Horsfield (1987)				2.54–2.81	1.5	1.55
Tawhai (2000)	1.39	1.109	1.14	2.358	1.323	1.344
Tawhai (2004)	1.47 (0.99)		1.13 (0.87)	2.8 (1.00)	1.41 (0.98)	1.39 (0.95)
Schmidt (2004)	1.48					

The data are given for our model and the models of Phalen et al. [36], Horsfield & Thurlbeck [50], Horsfield et al. [51], Tawhai et al. [28,31] and Schmidt et al. [52]. The R^2^ of the data is given in parentheses where available.

**Fig 4:**
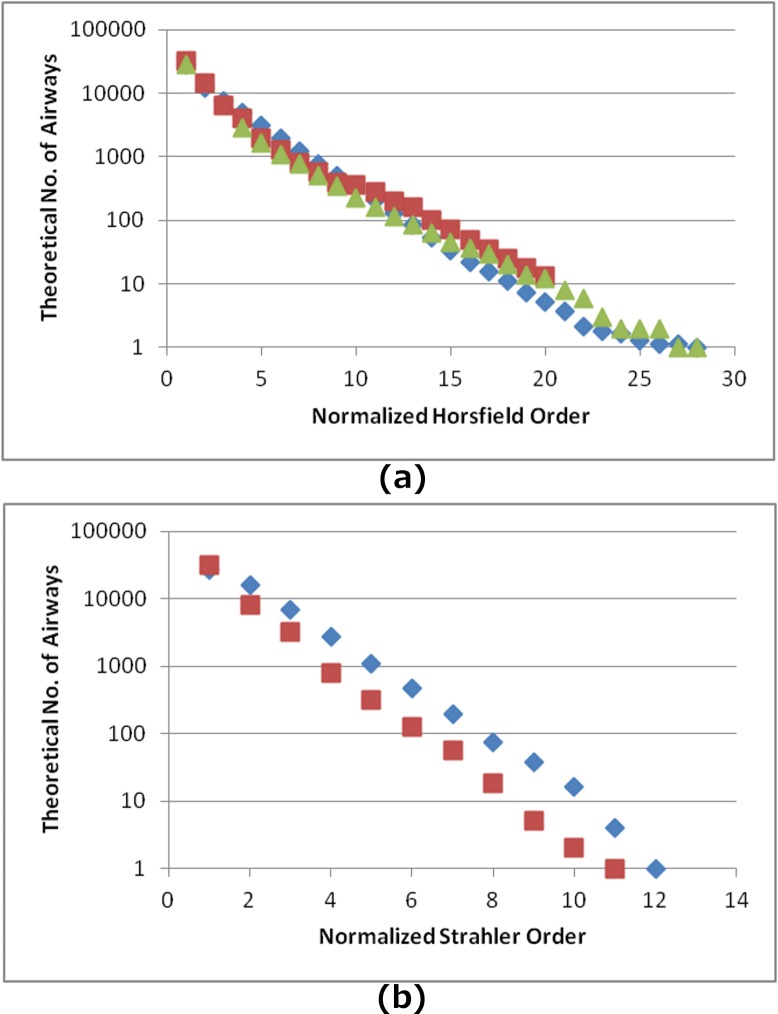
The interpolated number of airways per normalized a) Horsfield order and b) Strahler order for our model (blue ◆), the models of Tawhai et al. [28] (red ■) and Horsfield and Cumming [23] where available (green ▲). The vertical axis is shown in logarithmic scale.

Finally, the number of Horsfield (u_hMAX_ = 26, avg. 25) and Strahler (u_sMAX_ = 13, avg. 12) orders predicted by the model are lower) and higher respectively as compared to [23] (u_hMAX_ = 28), [28] (u_hMAX_ = 26) and [31] (u_sMAX_ = 11). Since an airway tree begins at the trachea and ends at the TB, for a meaningful comparison between the different datasets a normalization of range of orders needs to be made. Therefore, for each subject in our model an AGR of 28 (Horsfield) and 12 (Strahler) was considered and a theoretical number of branches was interpolated to fit the new AGR. The result is displayed in Fig 4A and 4B.

### Diameter

**Fig 5:**
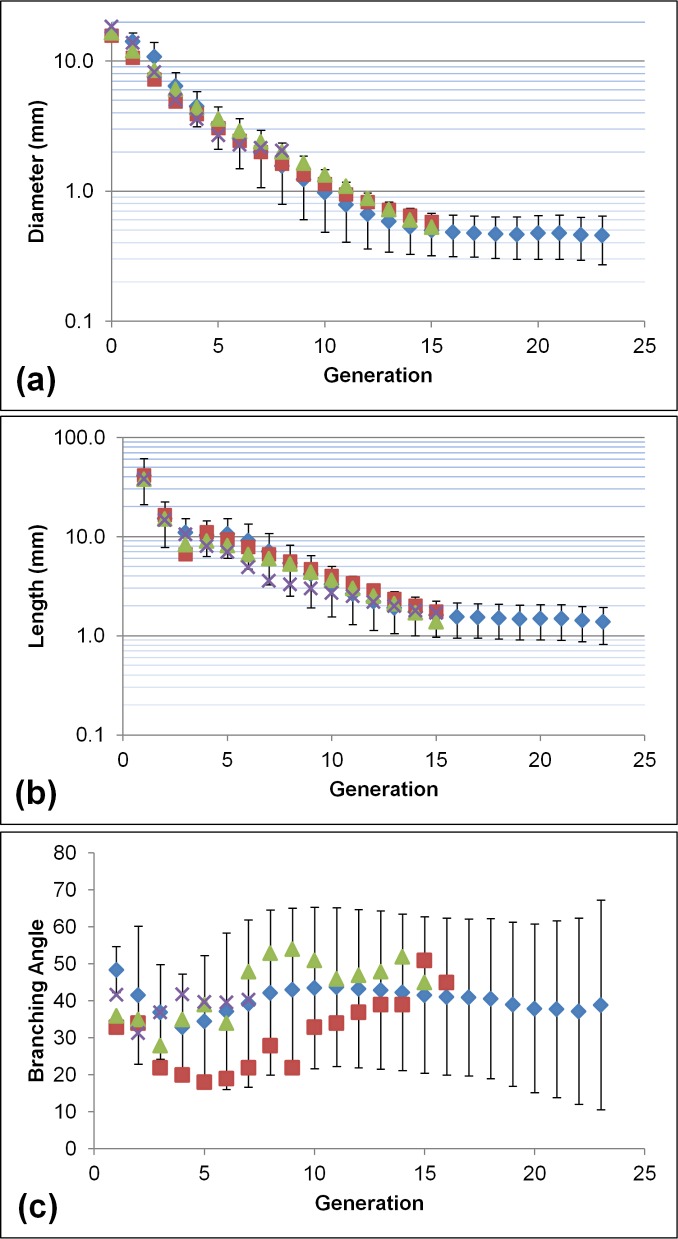
a) The diameter per generation D(j) for our model (blue ◆), the models of Soong et al. **[24]** (red ■), ICRP **[3]** (green ▲) and the data of Montaudon et al. **[53]** (purple x). The standard deviation obtained for our model is also displayed in the form of black limited lines. The vertical axis is shown in logarithmic scale. b) The length per generation L(j) for our model (blue ◆), the models of Soong et al. **[24]** (red ■), ICRP **[3]** (green ▲) and Phalen et al. **[36]** (purple x). The standard deviation obtained for our model is also displayed in the form of black limited lines. The vertical axis is shown in logarithmic scale. c) The branching angle per generation θ(j) for our model (blue ◆), the models of Yeh and Schum [1] (red ■), Phalen et al. [54] (green ▲) and Sauret et al. [55] (purple x). The standard deviation obtained for our model is also displayed in the form of black limited lines.

The average diameter for our model and three more models in the literature is plotted on log scale against generation in Fig 5A. It can be observed that the model is in good agreement with all three published datasets, especially between generations 5–10. Some differences exist in the first 4 generations where the greatest structural variability exists in the tree. Between generations 10–15 our model predicts slightly lower values. The difference with the literature is less than one standard deviation, with the diameters in the model remaining stable after generation 15, where the conducting part of the tree is usually considered to be replaced by the gas-exchange part.

The rate of decline of diameter with depth, given by the HR, was computed to be HR = 0.789±0.194, almost identical to the theoretical value calculated by [42,44] and confirmed experimentally in [22,31] but a bit lower than the one suggested by [56], where HR = 0.85. Montaudon et al. [53] found this value to vary between 0.65 and 0.97 while [34] measured HR = 0.72 for the first 6 generations. The HR for the major and minor daughters was measured to be HR_maj_ = 0.914±0.087 and HR_min_ = 0.663±0.184. The former is only marginally lower to the one predicted by Tawhai et al. [31] while HR_min_ is almost identical to the results of both [31] and [34]. Finally, in their implementation of the model, [32] calculated min and max HR of 0.81 and 0.85, with zero std.

Table 2 contains the diameter ratios for the model and other literature models. Data are scarce for the Horsfield ordering system, where the current study gives RD_H_ = 1.166, only slightly larger than the value of 1.109 given by [28]. RD_S_ is better documented, with the model predicting RD_S_ = 1.397, in the middle of the range 1.35–1.45 given by [50] and very close to 1.41 given by [31]. Phalen [36] and Tawhai [28] calculated slightly lower RD_S_ (1.351 and 1.323 respectively) while [51] found it to be slightly higher at 1.5.

Several methods have been used in the literature for the measurement of TB diameter (D_TB_). Some studies used models for predicting the D_TB_ using some particular termination criteria while others performed direct measurements on lung casts either manually or via medical image processing. In the former category, Soong et al. [24] working on data from [22] found D_TB_ = 0.58±0.29, Phalen et al. [54] calculated it to be 0.46 and Kitaoka et al. [27] computed a value of 0.48±0.06. The two models by Tawhai et al. [28,31] predict a D_TB_ of ~0.375 and ~0.6mm respectively, dependent on the conditions used to initialize and terminate their algorithm. Direct cast measurements made by [35] and [38] gave D_TB_ = 0.5±0.054 and 0.432±0.035 mm respectively while Litzlbauer et al. [37], using synchrotron CT in a limited set of images, measured D_TB_ = 0.66±0.04 mm. Using the specific set of stopping conditions mentioned earlier, the model estimates D_TB_ = 0.418±0.114mm, a value equivalent to [28,38,54] but relatively low compared to [24,27,31,35,37].

### Length

Fig 5B displays a log-normal plot of the airway length per generation for the model compared to three other models. The results are similar to the ones of diameter [Fig 5A], with the exception of the model of [36] in generations 5–10.

The length ratios for the model [Table 2] are RL_H_ = 1.13 and RL_S_ = 1.39 and are identical to the values calculated by Tawhai et al. [31]. RL_S_ is in the middle of the range 1.33–1.44 proposed by [50] and very close to the value of 1.44 and 1.33 found by [28] and [36] respectively. The highest value found in the literature is 1.55 [51].

An important relationship for our model is the one given by the ratio between airway length and diameter. In this study it was found to be 3.25±0.52, which is somewhat higher than expected since the value was set through Eq 4 and is obviously affected by the length and diameter constraints. It was also found to be higher than the average value 2.835±1.03 of [39], 2.92±0.92 of [31] and 2.72±1.43 of [34], all of which also exhibit much higher standard deviations, but lower than [32] who obtained a value of 4.51±0.25. A frequency distribution analysis of L/D indicates that 81% of the bronchi in our model are in the range 2.5–3.5, 15% between 3.5–4.5 and the rest between 4.5–5.5. No differences were found between major and minor branches.

Finally, the TB length (L_TB_) predicted by the model was L_TB_ = 1.34±0.44mm. Unlike D_TB_ the values for L_TB_ presented in the literature demonstrate considerable variability. Schreider and Raabe [38], Haefeli-Bleuer and Weibel [35] and Litzlbauer et al. [37] find L_TB_ = 0.819±0.14mm, 0.8±0.354mm and 0.93±0.43mm, respectively. However, other studies found L_TB_ closer to 1.8mm [24,31,54]. It has to be mentioned that the set of results with the lower values is obtained via direct measurements on TBs while the higher values are obtained through predictive models. Our result is close to the middle of the range set by the lower and higher values found in the literature, displaying a reasonable amount of variability and is close to the L_TB_ proposed by the ICRP model (1.38mm) [3] and Hansen and Ampaya’s measurements (1.2mm) [45].

### Branching Angle

**Fig 6:**
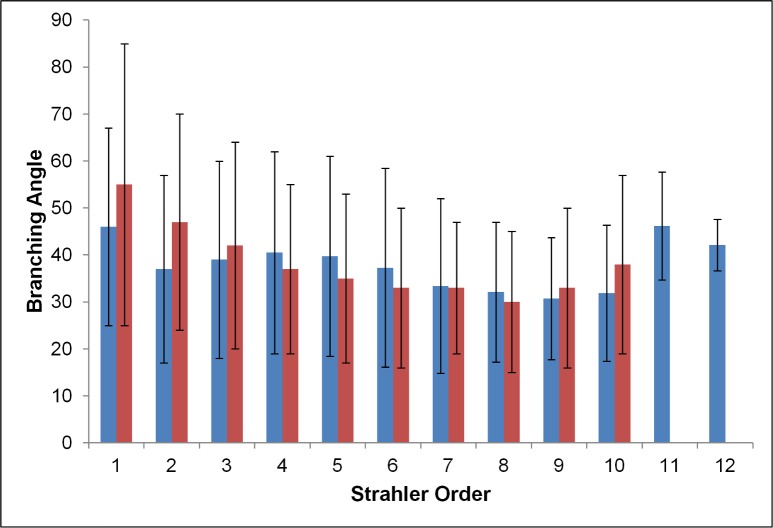
The branching angle as a function of Strahler order for our model (blue) and the model of Tawhai et al. [31] (red). The error bars are standard deviation.

There is a relative scarcity of data in the literature concerning the distribution of θ per generation [θ(j)]. Fig 5C displays the average θ(j) for the model and other reported data. It can be observed that the average value per generation is almost constant over the entire generation range but displays considerable variability, as indicated by its high standard deviation. The data of [54,55] are in general agreement with our average values; in fact, the only set exhibiting strong differences with the rest is that of [1]. For a more direct comparison with the model of Tawhai et al. [31], the θ was also plotted against u_S_ in Fig 6. The differences between the two models per order were not very large, with the exception of orders 1 and 2, where [31] predicts ~10° higher θ.

**Fig 7:**
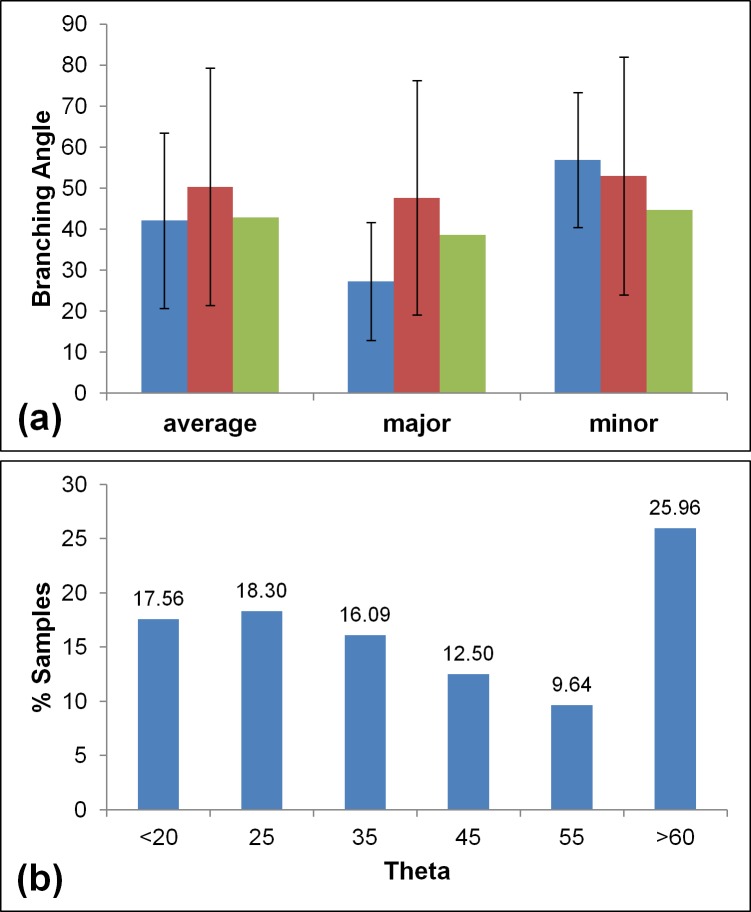
a) The average, major and minor child branching angle for our model (blue) and the model of Tawhai et al. **[31]** (red) and Bordas et al. **[32]** (green). The error bars are standard deviations. b) The statistical distribution of the branching angle in our model.

The average θ in our model is 42.1°±21.4°, close to the 43.4°±8.2° of the model proposed by the ICRP [3], and ~5^ο^ higher than the theoretical value of 37.28° [41–44], while [32] calculated θ = 42.89 but with almost no standard deviation. When compared to the data of [31,32] (Fig 7A), some marked differences can be observed, especially where the θ of the major child is concerned.

Due to the large variability demonstrated by our model the frequency distribution of the θ was calculated (Fig 7B). A relatively large number of samples (26%) is concentrated between 60° and 75°, indicative of the asymmetry in the bifurcation angle. It was computed that 15.4% of the samples reach the threshold value of 75°. If the fact that the major daughter has lower θ in 90% of the cases is considered, it can be confirmed that the range 0°-45° is dominated by the major child while 45°-75° dominates the θ of the minor child. This agrees with the model proposed by [57], where they found θ_maj_>θ_min_ at 75% of the TB tree samples and it signifies a high asymmetry to the branching of the conducting tree, possibly higher than that of [31,32].

### Planar Rotation Angle

**Fig 8:**
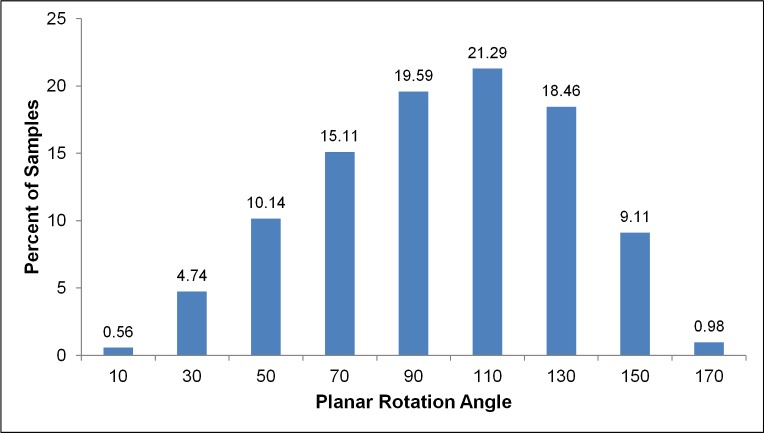
The frequency distribution of the planar rotation angle in our model.

The rotation angle φ was found to be 97.5°±33°, comparable to the value of 90° considered as optimal [26] and matching the models of [27,31,32] (90°±7°, 90°±43.3° and 90°±0° respectively). Measurements on the first 7 generations of the TB tree made through analysis of HRCT images [34] revealed a similar value of 88.1°±41.6°. The frequency distribution, displayed in Fig 8, reveals a slightly slanted Gaussian shape with samples throughout the range 0°-180°.

### Acinar Volume

**Fig 9:**
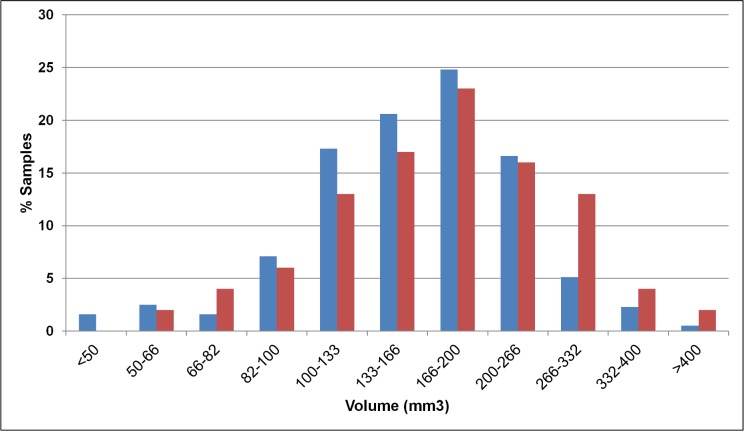
Frequency distribution of the acinar volume for our model (blue) and the results of Haefeli-Bleuer and Weibel [35] (red).

The V_ac_ was found to be 171.6±77.4mm^3^, very close to the literature data [35,37,45,46]. The results of a statistical distribution analysis were compared with an equivalent analysis from [35] and can be seen in Fig 9. Both distributions have similar shape with a maximum in the range 166-200mm^3^. Two important differences can be observed. The first one is in the range 266-332mm^3^ where our model predicts a number of airways ~8% lower than the experimental measurements. This is compensated for by the combined difference in the range 100-200mm^3^ where our model has ~10% more samples than [35]. The second difference is that our model estimates a small number of samples (~1.5%) with V_ac_<50mm^3^ while very few acini have volume higher than 400mm^3^.

## Discussion

Geometric models of the lung or its sub-structures have been used for the investigation of various issues, including the simulation of particle deposition patterns [2,7,11,58], the interpretation of functional medical images [4,6,18,58] and the prediction and explanation of regional ventilation information [13,15–18]. Even though all the above topics are valuable for the diagnosis and treatment of lung disease, most of the relevant studies found in the literature where airway tree models are employed concentrate on healthy subjects; indeed, even studies examining asthma [13,18] utilize models designed for the simulation of healthy air-trees, with the latter performing a scaling of airway diameter to replicate conditions of disease in their model. Furthermore, even though airway diameter was shown to be between 10 and 25% lower in asthmatic patients when compared to healthy subjects [59,60], this average value cannot accurately represent the great intra-subject variability in disease phenotype [14] or the various local bronchial constrictions that can affect airflow globally [34,60]. As a matter of fact almost all research concerning the changes in bronchial geometry due to disease remodeling in asthma and COPD concentrates on very specific features that include inner and outer diameter, lumen and wall area and their correlation to clinical outcomes such as FEV_1_, FEF_25-75_ or FEV_1_/FVC [20,61–64]. No information with regard to the effect of disease on bronchial length, number and location of airways in the lung, branching angles or even the volumes of acini could be found. In our opinion, all these characteristics and their respective relationships (L/D ratios, correlation between D and θ, the effects of gravity angle on airway and acinar shape etc.) are significantly affecting the shape of a diseased airway tree and should be much more thoroughly investigated and integrated into the predictive models currently used.

The airway measurements reported herein were made using a combination of the commercially available software packages described in the Methods and techniques developed by our group [34]. Several other methods and software for the segmentation and measurement of the respiratory tract are described in the literature [53,65–68]. As a matter of fact, most data available from previous studies on medical images were acquired using a variety of techniques and algorithms. However, the selection of the data acquisition technique usually affects the results of the analysis, with a most characteristic example being the process used for airway skeletonization. The airway tree skeleton is typically produced through erosion of the binary mask of the segmented airways, a process prone to artifacts but also heavily affecting the estimation of basic air-tree quantities such as diameter or wall thickness. For this reason an understanding of the different methods for the acquisition of morphometric information is essential if the resulting morphology models are to be meaningfully compared.

The HRCT images used as the basis for this study were captured during a complex breathing maneuver such that the relative volumes compared to FRC are variable. It is safe to assume that most morphometric parameters, including the lengths, diameters, hydraulic diameters and even the wall thicknesses are affected by the change in air volume during respiration. Unfortunately, not much direct information exists on the geometric changes to the airways that occur during a respiratory cycle. Some very limited work has been conducted for humans, concentrating on the differences in lumen area of specific airways in disease conditions [62,69] while similar work has been found with respect to Wistar rats [70]. More recently, Yin et al. [17] calculated the deformation of the segmented central airways between three levels of lung inflation but did not go as far as applying the deformation to the model, using it simply as an intermediate to tie the TB region ventilation fractions to the exits of the segmented bronchial airways as boundary conditions for CFD simulations. This raises the important question concerning the overall behavior of the bronchial tree in 4D, chiefly with regard to the geometric relationships used, especially in the presence of disease.

When investigating the airway tree, one of the biggest difficulties is comparing branches that perform similar anatomical functions. In our study, comparisons were made using three popular classifications. However, all three systems suffer from the same disadvantage, which is practically the very large inter-subject variability within species, especially in the central lung regions. Both Horsfield and Strahler ordering systems are viable options when the deeper lung is concerned but the large asymmetry in the central tree suggests that a normalization in the order range is necessary prior to data comparison. Therefore, even though the branching, diameter and length ratios (RB, RD, RL) are good measures for the prediction of the basic geometric characteristics of the human lung (including symmetric properties), they cannot give us the entire story. This is evident through the discrepancy of the two plots in Fig 4B, where the RB_S_ between datasets is similar.

The algorithm implemented in this study is based on the algorithm proposed by [28] and has many common features with the work of [32]. However, some marked differences also exist. The diameter of the generated airways was assigned using a relationship with the airway length instead of either a parent-child relationship or an assignment based on lung depth (generation or order). Our results show very good agreement to previous experimental and modeling data for a healthy lung, with a moderate amount of variability as is expected in complex biological systems [56]. Some other differences with [28,32] include the use of segmented volumes rather than surfaces as confining spaces, the use of multiple seeds per acinus, which allows for a variation of V_ac_ as a stopping condition and also brings the shape of distal sub-spaces into consideration, and the recursive re-assignment of seeds during the formation of airways. The latter, along with the selection of splitting plane, seems to be the main reason for the more realistic angular variations present in this model, as opposed to the model of [32] where both θ and φ show zero variability.

Several important improvements are possible for the model. The influence of sub-space shapes during the generation of the tree skeleton could be included. The iterative redistribution of the sub-volume seeds in the case where the tree expansion stops under the condition of TB length; this should eradicate the very few, but still important unrealistically large acini predicted by the model (Fig 9). Furthermore, the parameters affecting branching and geometric symmetry could be examined and configured for the adaptation of the model with respect to species (increased symmetry vs monopody) [71]. Additionally, the incorporation of features of disease, such as the bronchial cross-section shape and perimeter or radius of curvature, as well as variations referring to respiratory motion would be necessary for the model to provide more precise calculations for diagnosis and therapy. The stopping conditions (L_TB_, V_ac_) could also be modified to dynamically take into account the stage of the respiratory cycle of the model starting conditions, i.e. the medical images. Finally, the current format of the model does not take into account the volume already occupied by the “real” tree, either the segmented version obtained by the HRCT images or the more proximally located model data, the latter being 1D prior to a diameter being assigned to them. This final correction is designed to remove seeds from the “base” of the confining spaces, thus reducing the local branching angles.

In conclusion, in this study the detailed implementation of an algorithm was presented for the generation of a 3D model of the human trachea-bronchial tree. A series of tree structures based on this algorithm were then generated using a set of HRCT medical images of healthy volunteers. This was followed by a thorough statistical analysis of the results and the evaluation of the realism of the models by comparison with measurements and other model predictions from the relevant literature, which was extensively reviewed for this purpose. The current model appears to agree very well with most previously reported data, both with respect to its morphometric characteristics but also in connection to the relationships connecting different geometric features such as the HR or the L/D ratio. It has to be mentioned that the validity of many interesting attributes observed in the model could not be confirmed due to a lack of equivalent morphological studies in the past. Finally, a set of improvements for the model were proposed, the most important of which involve the adaptation of the process to multiple species and conditions of disease. Still, as has been proposed before [16,17,63], the model can be used in its current form for the preliminary interpretation of functional medical images and the prediction of gas flow and aerosol deposition patterns.

## Supporting Information

S1 File Model Morphology Data.zip. A set of two files per subject containing the morphology and connectivity information of the airway tree (both “real” and “model” data). The _morphology.txt file includes the total lung volume of the subject lung in cm^3^ and the %volume per lobe. It also includes the following fields in each row (one row per airway): (i) *Vida_Name*: The name assigned to each airway by the Vida PW2 software (0 if none exists), (ii) *CenterLineLength*: The distance between airway start and end coordinates in mm, (iii) *Diameter*: The airway average diameter in mm, (iv) *Branching_Angle*: the airway branching angle in degrees, (v) *VidaGeneration*: The generation assigned to each airway by the Vida PW2 software, vi) *xs*, *ys*, *zs*, *xe*, *ye*, *ze*: The coordinates of the start and end node of each airway in voxels, (vii) *Adept_Names*: The individualized naming scheme for each airway; in-house terminology is used, (viii) *Generation*: The airway generation assigned to each airway using a Boyden-based scheme, (ix) *RootFlag*: A flag indicating whether the airway is a “leaf” in the tree (1) or just a tree branch (0), (x) *SpaceVolume*: The volume of the space in the lung supplied by each airway in mm^3^. The translation.dico file contains information on airway connectivity, explicitly stating the parent-child relationships in the tree.
(ZIP)
